# Retained non-previa placenta in the era of “placenta accreta spectrum”: a report of two cases managed expectantly and a proposed plan for management

**DOI:** 10.3389/fmed.2025.1504491

**Published:** 2025-04-28

**Authors:** Mohamad K. Ramadan, Nouhad El-Zein, Murchida Jomaa, Abir Zeidan, Rana El Tal, Dominique A. Badr

**Affiliations:** ^1^Division of Maternal-Fetal-Medicine, Department of Obstetrics and Gynecology, Makassed General Hospital, Beirut, Lebanon; ^2^Department of Obstetrics and Gynecology, Makassed General Hospital, Beirut, Lebanon; ^3^Department of Obstetrics and Gynecology, University Hospital Brugmann, Université Libre de Bruxelles, Brussels, Belgium

**Keywords:** expectant management, placenta accreta spectrum, placenta adherent, ultrasonography, retained placenta

## Abstract

Retained placenta (RP) is the absence of placental expulsion within 30 min of neonatal delivery. It is an obstetric complication affecting 0.5–4.8% of all vaginal deliveries. We report two cases in which the patients were primiparous. Patients were initially kept at the hospital under close observation. The lack of spontaneous detachment and the absence of bleeding prompted us to resort to an expectant approach approved by both patients. A decrease in B-hCG levels was followed by a steady decrease in placental size and the resumption of regular menses. The management of RP should be individualized according to hospital resources, patient fertility desire, sonographic characteristics, the presence of hemorrhage, and hemodynamic stability. RP should prompt the mobilization of resources needed for managing postpartum hemorrhage (PPH), which might ensue without notice. Manual removal of the placenta (MROP) has been recommended for managing RP regardless of hemorrhage or retention etiology. MROP, however, might initiate massive bleeding, infections, prolonged hospitalization, the need for curettage and hysterectomy. Moreover, if MROP is attempted in an unidentified placenta accreta spectrum (PAS), it might initiate life-threatening hemorrhage, necessitating the performance of hemostatic interventions, including emergent hysterectomy. Serious considerations should be given to mitigate the indiscriminate use of MROP in the era of the “PAS epidemic.”

## Introduction

Retained placenta (RP) is defined as the lack of placental expulsion 30 min following active management or 60 min following physiological management of the 3rd stage of labor (1). It is estimated to complicate 0.5–4.8% of vaginal deliveries (2). It is more common after preterm birth (9.1%), whereas at term, it affects only 1% of the population (3). If it is neglected, it can lead to postpartum hemorrhage (PPH) or endometritis (4). RP is associated with 20% maternal mortality due to severe PPH (5). There are many well-defined risk factors, including a history of previous RP or manual removal of the placenta (MROP), uterine surgery, uterine anomalies, preterm delivery, prolonged oxytocin use during labor, assisted reproductive technologies (ART), stillbirth, advanced maternal age, and multiparity (2). Recently, with the increasing popularity of conservative management, placenta accreta spectrum (PAS) has also become a novel risk factor (6). In RP associated with PPH, the first recommended action is MROP (7), whereas without bleeding, there are no clear guidelines for optimal treatment (8). Nonetheless, the consensus view advocates MROP when RP persists beyond 30 min of PPH (9). MROP is not always without consequences; it is an invasive procedure that can lead to massive hemorrhage, hemodynamic instability, and the need for emergency interventions, including blood transfusion, interventional radiology, curettage, endometritis, and hysterectomy (10, 11). Moreover, in the presence of an undiagnosed PAS, extirpation or forceful extraction of the placenta can lead to massive and potentially catastrophic PPH and unplanned emergency hysterectomy (12). Although ultrasonography can accurately identify the cause of RP, it has a low detection rate for “non-previa PAS (13, 14). The management of RP continues to be one of the most difficult challenges in obstetrics (15). Herein, we report the clinical course of successful expectant management of retained adherent fundal placentae in two primiparous patients. This is followed by the proposal of an ultrasound-based stepwise plan for managing RP.

## Description of the cases

### Patient 1

**Figure 1:**
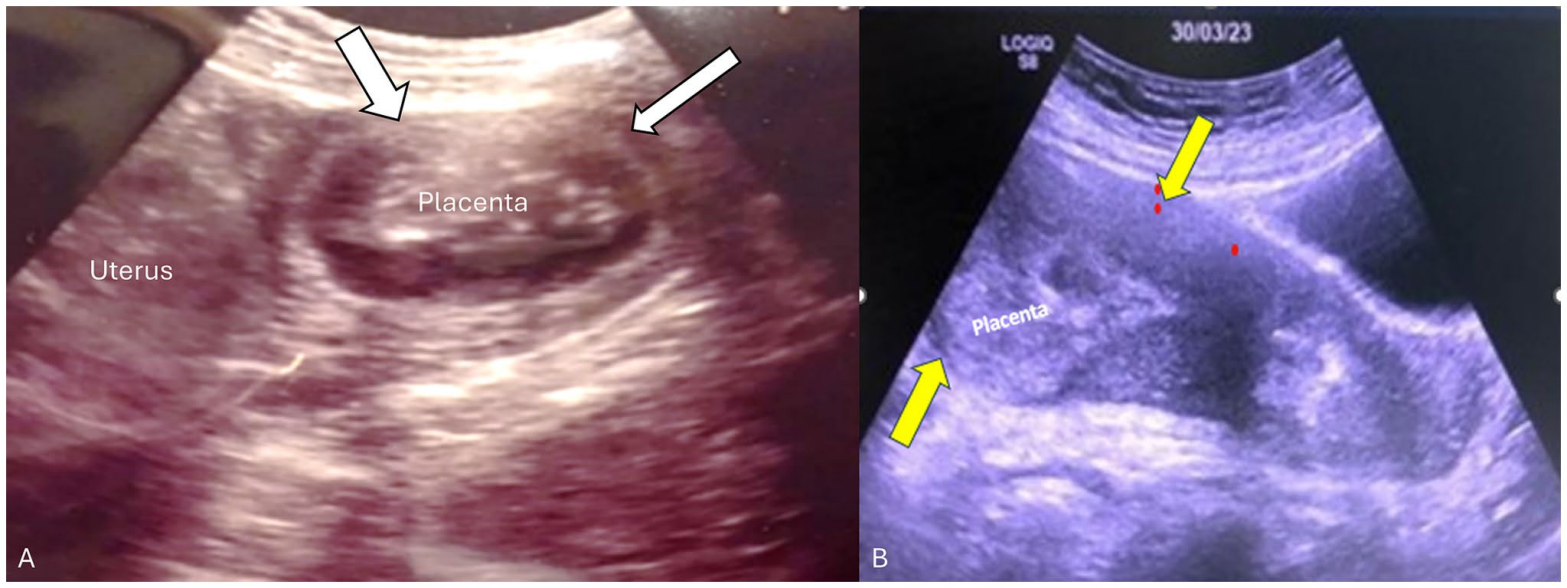
Transabdominal ultrasound of the placenta and uterus in both patients. **(A)** Transverse view of the uterus showing the placenta in the left cornua 14 weeks postpartum. Myometrial thinning was seen in the fundal portion adjacent to the placenta consistent with placenta accreta/adherent (white arrows). **(B)** Sagittal view of the uterus showing retained fundal placenta. Yellow arrows demonstrate a thick contracted anterior-wall myometrium whereas the posterior uterine wall is very thin (non-detached partial placenta adherent).

This was a 30-year-old obstetrician, G2P0A1, with a non-significant history except for a first-trimester spontaneous abortion that was treated medically. During the index pregnancy, she presented at 31 weeks gestation with active labor a few hours after premature rupture of membranes. Cardiotocogram (CTG) revealed the presence of active labor and recurrent fetal decelerations. An urgent cesarean delivery was performed and led to the delivery of a live newborn female weighing 1,400 grams. The placenta was not delivered spontaneously. After exteriorization of the uterus, a large myometrial bulge was noted at the left fundal cornua. PAS was highly suspected, and the decision was to keep the placenta *in situ*. She was started on broad-spectrum antibiotics and was kept at the hospital under close observation. The patient, as herself an obstetrician, requested to be treated with methotrexate. This agent was administered once weekly (1 mg/kg) until the disappearance of B-hCG. Follow-up was performed with complete blood count, C-reactive protein, and imaging (Figure 1). The earliest sonographic feature was the cessation of vascular flow around and inside the placental mass, followed by a steady decrease in the placental mass. Several weeks later, she reported an episode of transient vaginal bleeding requiring 24 h of in-hospital observation without blood transfusion. Six months later, the patient reported the passage of an oblong grayish finger-like mass. Soon after, she started to have regular menses. She conceived 7 months after the restoration of regular menstruation and gave birth to a full-term infant after an uneventful repeat cesarean delivery without PAS or recurrence of RP.

### Patient 2

**Figure 2:**
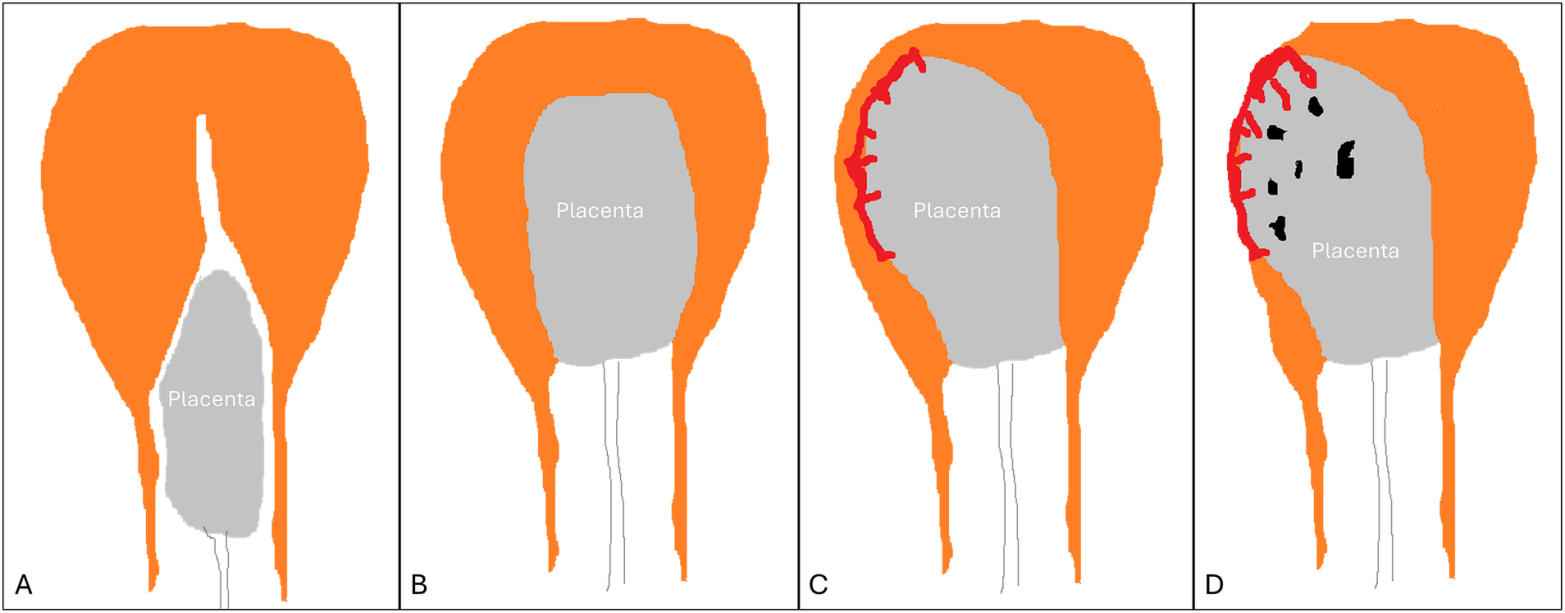
Types of retained placenta. **(A)** detached yet entrapped placenta. **(B)** normal placenta undergoing detachment (incomplete detachment). **(C)** non-detached adherent placenta (normal or placenta accreta spectrum). **(D)** placenta accreta spectrum.

This was a case of a 36-year-old female, G1P0A0, who delivered vaginally at 17 WG in a nearby maternity. She had no relevant medical or surgical history. The patient experienced preterm premature rupture of the membranes followed by spontaneous labor and delivery. Many attempts to deliver the placenta resulted in avulsion of the umbilical cord, so she was referred to our department 1 hour after delivery. At presentation, the physical examination revealed stable vital signs without vaginal bleeding but with a closed cervix. An ultrasound scan revealed a heterogeneous mass of 4 × 5 cm at the uterine fundus (Figure 2). The overlying myometrium was very thin on one side, but blood flow on color Doppler was absent. A diagnosis of a retained partial placenta accreta/adherence was made. Blood tests revealed the following results: hemoglobin (Hb), 11.6 g/dL; platelet count, 329,000/μL; white blood count (WBC), 18.8/μL; CRP, 8.5 mg/L; and a normal coagulation profile (international normalized ratio, 1; activated partial thromboplastin time, 35.5 s; fibrinogen, 488 mg/dL).

She received one dose of prophylactic intravenous antibiotics, which was later switched to an oral route for 1 week. Since there was no bleeding and no clinical or sonographic features of placental separation, an expectant approach was applied. The plan was explained to the family, and written consent was acquired. Misoprostol (400 μg) was started every 4 h for 5 doses, but this did not result in placental expulsion or cervical changes. Her WBC and CRP levels returned to normal 5 days later, so she was discharged home. The patient was seen weekly for the first 2 months. The visits included clinical assessment, ultrasound scan, and laboratory screening for infection. She was also requested to report any foul-smelling discharge or vaginal bleeding. Beta-hCG was 40 IU/dL on day 8 and became negative 3 months later. During follow-up, she was stable, with no documented fever. Pelvic ultrasound after 1 month revealed a decrease in the size of the placenta to 3×3 cm, with areas of degenerative necrosis and the absence of blood flow inside and around the placenta; the surrounding myometrium became thicker. Three months later, ultrasonography revealed significant resolution in placental dimensions of 1.1 cm × 1.2 cm, without vascular activity. The most recent transvaginal ultrasound revealed complete resorption of the placenta and a thin, regular endometrium. She started having regular menses 3 months postpartum, and she is currently contemplating a new pregnancy.

## Discussion

Active management of the third stage of labor (AMTSL) can effectively decrease the frequency and severity of primary PPH; however, it does not impact the RP rate or the need for MROP, even with the addition of placental drainage (16). This indicates that RP might not be predictable or preventable (2).

Two main forms of RP are substantiated according to sonographic findings (17), in addition to a third transitory form briefly seen during the process of expulsion (Figure 2):

1. An entrapped yet completely detached placenta. This might be secondary to structural uterine anomalies, maternal exhaustion, premature cervical closure, or a constriction ring in the lower uterine segment hindering the free descent of the placenta into the lower birth canal before its expulsion. Here, the placenta can be seen by ultrasound in the cervical canal or the upper vagina, whereas the uterine fundus is empty with a thick (contracted) apposing myometrium.
2. A normal placenta in the process of detachment but with a protracted slow progression: Sonography reveals a placenta surrounded by thick myometrium but is still high inside the uterine cavity. This sonographic appearance is transient and short-lived during the passage of the placenta through the birth canal.
3. An adherent placenta with a thin underlying myometrium in the placental bed, while the remaining uterine walls display a thick myometrium, creating a feature of myometrial asymmetry. These features might be limited to a small area or involve the entire placenta. This entity can be caused either by an adherent normal placenta or by any form of PAS. It is believed to be secondary to weak myometrial contractions or to anchor villi situated deep into the myometrium, preventing its effective contraction.

Most cases of RP are of the “adherent placenta” type. Using ultrasound, it is difficult to discriminate between a non-PAS-adherent placenta and a PAS-adherent placenta (13, 18). Shapiro et al. (19) described a case of RP where sonography revealed extreme myometrial thinning. Compared with that in the adjacent portions of the uterus, the myometrial thickness was markedly asymmetric, with significant thinning in the placental bed. The patient developed endometritis that mandated hysterectomy (19). Petrovic et al. (20) described a case of an adherent placenta that was successfully removed surgically (piecemeal) under real-time ultrasound control after PPH and failed MROP. Jauniaux et al. (21) described another case of non-previa accreta where antepartum sonography revealed extensive myometrial thinning limited to a focal area. Delivery was complicated by bleeding of the partially retained placenta. Bleeding was treated by resection/reconstruction of a focal segment and the use of hemostatic sutures (21). Histopathologic examination of the 3 patients revealed either focal or total PAS. Myometrial thinning at the level of the placental bed was a common sonographic feature in the three patients. Previously, it was also used by Herman et al. (22) to identify cases of adherent placenta; nonetheless, this sonographic feature seems to be common to adherent normal placenta and PAS.

Most non-previa PAS are not diagnosed prenatally and are only encountered during delivery as a retained placenta or following hemorrhage during attempts at manual removal (13). Placenta accreta accounts for 8.5–50% of the retained placenta (13). A non-previa PAS continues to present a diagnostic challenge. Only 29% of patients present with the sonographic features used in the diagnosis of previa PAS (13). Unlike previa-PAS, there is no standardized, well-structured protocol for sonographic imaging of non-previa PAS. Hence, placental tissue that fails to separate after delivery may fit within the PAS (17). The resorption events observed with RP are also observed with PAS when treated conservatively (23). Therefore, extrapolating the experience attained with the conservative management of PAS can be valuable in the management of RP. In the absence of reliable imaging methodology to differentiate between adherent normal placenta and adherent PAS, the duration of placental retention can be used to distinguish between placentas. Longer retention favors true accreta, whereas spontaneous separation in less than 24 h is usually associated with a normal adherent placenta. Jauniaux et al. (24) suggested excluding cases where separation occurs spontaneously between 30 min and 24 h, thereby mitigating false positive cases and excluding any confusion.

The identification of placental retention should prompt immediate preemptive steps in anticipation of PPH that can ensue without notice, particularly among at-risk parturients (4). These preparations include typing-cross-matching, assembling the PPH team, a readily accessible PPH box including uterotonic medications, balloon tamponades, and coordination with an anesthetist. If delivery occurs at a maternity or a small peripheral hospital with limited resources, transferring the patient to a higher-level hospital might be safer.

Previously, postpartum manual exploration of the uterine cavity was used to identify the presence of RP, and it was common to perform MROP to assess the presence of a cleavage plane between the RP and the underlying uterine myometrium. When this was not elicited, it was interpreted as invasive placentation, whereas when such a plane was easily created, it was construed as an adherent and noninvasive placenta (4, 22). This concept was also used by Collins et al. (25) to construct a clinical score for grading PAS in the absence of surgical specimens (48). This procedure, however, could be dangerous and should be avoided, as it would initiate massive bleeding. Furthermore, the risk of bleeding is believed to increase with increasing duration of placental retention (15, 26). This view has led many authors to interfere with immediate MROP following any delay in placental expulsion. Others, however, debate the idea of increased risk for PPH with longer retention times and deny justification for an early cutoff for MROP (5). Given the contemporary surge in the PAS rate, the indiscriminate employment of MROP to all RPs must be re-evaluated. Even in the presence of significant PPH, MROP can aggravate bleeding in the presence of invasive forms of PAS. Given the low accuracy of sonography in depicting non-previa PAS, it might be better to avoid MROP in cases of high clinical risk for PAS (13). Instead, it might be safer to proceed directly to hemostatic surgery or embolization in hospitals with appropriate settings or to employ tamponing balloons or intrauterine packing before transferring the patient to a specialized center with experience in PAS surgeries (27).

Conservative management of PAS aims at uterine preservation but can include auxiliary interventions such as embolization, placenta left *in situ*, uterine balloon tamponade, and methotrexate (28). Expectant management, on the other hand, entails leaving the placenta either partially or fully in situ and waiting for spontaneous resorption or expulsion (29). Conservative or expectant management approaches have recently gained increasing popularity not only for preserving the uterus but also when the surgical risk of cesarean hysterectomy is high (30, 31). When conservative management is employed for previa-PAS, it is associated with morbidity rates of 56–87.5% and various serious complications, such as late postpartum hemorrhage, infection, sepsis, DIC, delayed hysterectomy, uterine arteriovenous fistula, choriocarcinoma, and death (29, 30). Furthermore, this approach mandates meticulous and close observation of these patients for several months, pending complete resorption of the placenta. Moreover, women should be counseled extensively about unpredictable outcomes (32). Nevertheless, this management approach could successfully retain fertility potential and prevent hysterectomy in 78.4% of women (33). Live newborn delivery could be achieved in 92.5% of cases (34).

Adherent placenta seems to be equivalent to placenta accreta (FIGO PAS G-1), where there is no real invasion of the myometrium, and the abnormality is limited only to strong adherence of the placenta, which is believed to be due to the presence of a fibrinoid layer acting as a glue between the placenta and its myometrial bed (35). Morbidity and success rates seem to change according to the grade of “PAS,” with higher morbidity and less success with greater invasion, as seen in percreta (33). Consequently, more favorable results can be anticipated with less invasive forms. Nevertheless, no guidelines exist on the expectant management of non-previa PAS, including patient selection, the optimal strategy of clinical monitoring, or the success rates needed for patient counseling (23). The consensus view is to individualize the management approach according to patients’ characteristics. Furthermore, keeping patients with RP hospitalized for a few days under close observation can distinguish between those with significant adherence and those with nonsignificant adherence, which might resolve spontaneously within the first 24 h. Following this initial close-observation period at the hospital, patients are counseled and provided with information about available options for RP management.

Both cases described in this report share several clinical and sonographic aspects. Both were primiparas and developed RP following preterm birth. The first patient was a primipara with no prior uterine procedures. Her obstetric history revealed a 1st-trimester spontaneous blight ovum treated with misoprostol. Her current pregnancy was complicated with PPROM and preterm delivery with a firmly attached placenta visually estimated during cesarean delivery to be a retained placenta increta (grade 2) according to the score suggested by Collins et al. (25). The second woman was referred to our maternity service 1 hour postpartum after a failed MROP. The diagnosis relies on the presence of sonographic evidence of extreme myometrial thinning in the placental bed as opposed to the thick myometrium in the remaining uterine walls. Both patients were managed conservatively without major complications. In both cases, the sequence of events was as follows: cessation of placental bed vascular flow, gradual fading of B-hCG, resumption of regular menstrual cycles, and finally complete disappearance of the RP. The time lapse until complete resorption of the RP was shorter in patient 2, reflecting the smaller placental size. This observation was reported earlier by Fujishima et al. (36). Patient 1 conceived a few months later and gave birth to a full-term healthy newborn infant following an elective repeat cesarean delivery, without recurrence of RP or PAS.

## Proposed stepwise plan for RP management

**Figure 3:**
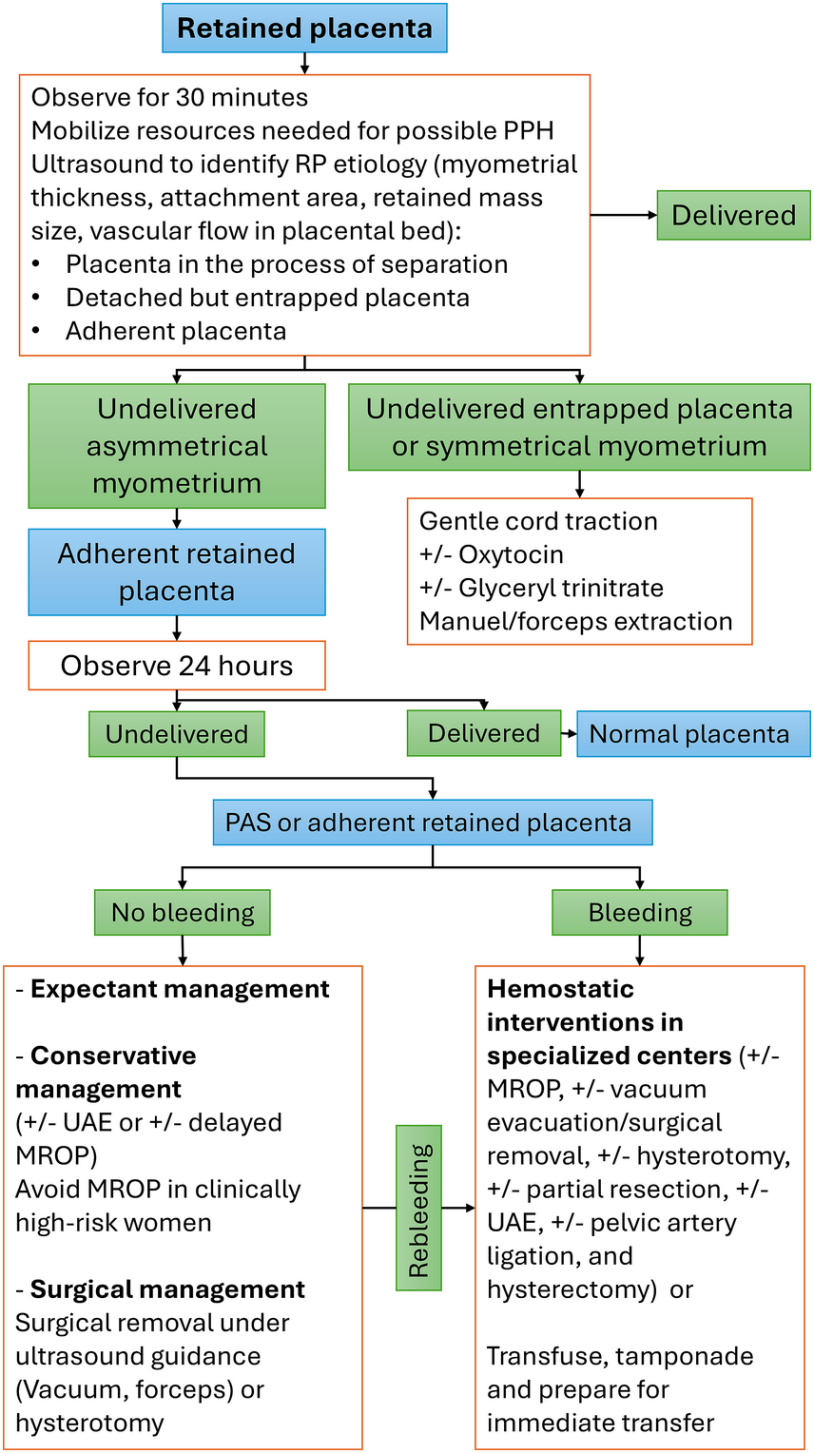
Stepwise management plan for retained placenta. MROP, manual removal of the placenta; PAS, placenta accreta spectrum; PPH, postpartum hemorrhage; RP: retained placenta; UAE, uterine artery embolization.

The conservative management of RP, including adherent placenta, consists of waiting for spontaneous expulsion without surgical intervention (36). Despite many reports providing robust evidence on the efficacy and safety of conservative management (36), several aspects and details of optimal patient counseling remain unclear. These include patient selection criteria, success prognostic factors, risk factors for hemorrhage or infection, and, most importantly, a detailed plan for the clinical, laboratory, and imaging surveillance of selected cases (Figure 3) (37).

### From 0 to 30 min after delivery

RP should be considered an obstetric emergency with an increased risk for PPH. Accordingly, appropriate preemptive measures necessary for managing PPH should be implemented immediately. This preparation should be completed before the first 30 min when the likelihood of PPH becomes substantially high (15, 38). The concept of increased risks of bleeding beyond the 30-min mark has been debated by a recent large study that reported no such association (5), yet preparing for possible PPH is a plausible idea. When delivery occurs at small maternities without access to blood transfusion services or the ability to perform hemostatic interventions, arranging for immediate patient transfer to a higher-level hospital might be safer.

### From 30 to 60 min

Ultrasonography is essential for monitoring and assessing the myometrial thickness of the surrounding uterine walls, vascular flow at the placenta bed, and retained mass size. Cases manifesting cessation of vascular flow and uniform myometrial thickening of surrounding uterine walls are anticipated to detach in a short period of time. In the cohort reported by Dombrowski et al. (39), spontaneous expulsion of the RP took place in 20–30% of these cases between 30 and 45 min, and an additional 10–20% of cases between 45 and 60 min, whereas no cases of spontaneous placental delivery occurred beyond 60 min. However, in another study, an estimated 50% of the remaining RPs showed spontaneous placental expulsion during the next 60 min postpartum (26). In the absence of bleeding, the use of uterotonic agents in managing RP has been the subject of immense controversy. Despite the lack of evidence to support the recommendation of uterotonics, additional oxytocin (10 IU, IV/IM) combined with controlled cord traction is recommended, which is expected to result in the expulsion of 90% of RPs within 60 min (40). The same guidance cautioned against the utilization of ergometrine (40). In another study, compared with expectant management, misoprostol did not improve the rates of placental expulsion or reduce bleeding rates or the need for MROP (26). Nevertheless, misoprostol 600 mcg orally or 800 mcg rectally has been used as a secondary option when oxytocin fails to affect placental expulsion (40). Different underlying etiologies can cause RP; hence, management should be individualized, and MROP might not be appropriate for all types of RP. When RP is associated with PPH, an ultrasound examination should also be performed to exclude the presence of PAS before the use of MROP; however, ultrasonography might not be decisive in non-previa PAS. A safer approach would be to avoid the use of MROP in the presence of non-previa PAS clinical risk factors (13). This intervention should be a part of a bundle of hemostatic procedures performed in the operating theatre after appropriate preparations (blood and a multidisciplinary hemorrhage team).

### Between 60 min and 24 h

Most RPs undergo spontaneous separation within the 1^st^ two postpartum hours, and only a few cases persist beyond the first 24 h. We believe that regardless of the underlying etiology of placental retention, any RP for >24 h can be managed the same way. Consequently, the methodology embraced in the conservative/expectant management of PAS can be of great benefit in the management of RP. This period can spontaneously distinguish between the adherent normal placenta and an adherent PAS G-1 (Accreta), where the former tends to detach within the first 24 h (24).

### Beyond 24 h

In the presence of sonographic features suggestive of invasive “PAS,” management is better achieved by a multidisciplinary team at specialized centers.

Intrauterine balloon tamponade was used to stabilize the patient and to decide on a management strategy or transfer the patient to a tertiary care facility (18).

Conservative/expectant management of RP might be complicated by intrauterine infection, the need for MROP, delayed hemorrhage mandating the use of hemostatic interventions (UAE, D&C, and hysterectomy), ICU admission, and possibly massive blood transfusion. Initial observation (1st few days) is better achieved at tertiary-care hospitals before discharge. Later, follow-up must also be achieved in coordination with a hospital that can provide immediate hemostatic surgeries and blood transfusions. These complications, however tend to be rare beyond 60 days postpartum (36).

The reported frequency of bleeding varies between 29 and 61%, according to different reports (17, 26, 36, 41, 42). Bleeding risk factors have been extensively explored and include larger RPs (17, 41, 42), ART (17), hypervascular RP (17, 42), the persistence of a feeding vessel (43) and delayed elective MROP (36).

The frequency and details of follow-up visits remain at the discretion of the caring obstetricians. In the study reported by Sentilhes et al. (33), the patient was called weekly for outpatient clinic appointments for the first 2 months. If asymptomatic, she will be seen monthly until the placenta has been completely resorbed. A clinical assessment (bleeding, fever, and pelvic pain), a pelvic ultrasound (size of retained tissue), and a laboratory screen for DIC (44) and for infection (hemoglobin and leukocytes, C-reactive protein, and vaginal sample for bacteriological analysis) were all part of the appointments (45). Complete and spontaneous disappearance of RP ranged between 50 and 100%, with a median time of 130 days, where the size of the RP plays an important role (21, 26, 36, 41). These figures are comparable to the rates of spontaneous and entire reabsorption observed after intentional conservative management of PAS, where the median was 13.5 weeks in 75% of cases (45).

Conservation of fertility potential with preservation of the uterus was possible among most women managed with a conservative/expectant approach. The utilization and choices of antibiotics can differ among centers, yet all patients receive broad-spectrum IV antibiotics for variable periods. The frequency of intrauterine infection was estimated to be 7%, whereas it was 9% with conservative management of PAS (17, 45). Notably, endometritis is uncommon beyond 60 days postpartum and can develop despite antibiotic prophylaxis for unclear causes (36).

Laboratory parameters are consistently monitored, although the frequency might vary among reports. These parameters are intended to monitor the development of anemia, infection, and coagulopathy. The B-hCG level is serially monitored until unmeasurable, despite a poor correlation with the placental size retained. It is expected to reach undetectable levels in 6 weeks but might vary according to the initial placental size (gestational age). Some centers consider the plateauing of B-hCG to be an indication to administer methotrexate with no scientific evidence of the efficiency of this approach. RCOG and FIGO do not recommend routine methotrexate use (9, 16). Furthermore, the B-hCG level is used to predict bleeding during delayed MROP but is not accurate in some cases. Nonetheless, levels tend to be low before spontaneous placental expulsion (41). Exceptions of this relationship can still be observed in patients with low or negative B-hCG levels who experience massive bleeding. Fujishima et al. (41) determined that it is difficult to determine whether conservative care would be successful based on the B-hCG level. B-hCG levels were not found to correspond with the volume of residual tissue (23). Thus, cases demonstrating progressively decreasing levels can be reassuring for a normal course. B-hCG tended to disappear at a median of 67 days among patients on conservative management (36). Regular menstrual cycles might resume even before the complete resorption of RP, and this was observed to correlate with the decline in B-hCG (23). This usually occurs at an average of 6 months postpartum, depending on the initial placental size/gestational age, even before the complete disappearance of the entire RPOC.

Conservative management has sometimes been complemented with interventions such as delayed MROP, surgical excision (D&C), hysterotomy, or hysteroscopic excision of placental parts to treat bleeding or to shorten the recovery time and decrease infection risk (30, 34, 46). These interventions might trigger massive hemorrhage, mandating radical hemostatic surgeries, including hysterectomy. These are justified in cases of rebleeding during conservative management (34) but otherwise are not warranted (36).

Women should be counseled about a negligible impact on future fertility (34) and a high success rate of liveborn delivery (34, 45). The recurrence rate varies according to the underlying etiology of RP, being 12.5% for non-PAS adherence (2) but 22.8–29% for PAS adherence (45, 47). Some authors recommend cesarean delivery in subsequent pregnancies, yet conclusive evidence is lacking (26).

The prognosis following conservative management of non-previa PAS seems to be more favorable than that following conservative management of previa PAS. PAS without previa was associated with a lower risk of invasive placenta, less blood loss, and less hysterectomy but was more difficult to diagnose prenatally (14). These facts can be extrapolated to support the conservative management of an adherent fundal RP.

## Conclusion

The cases described above demonstrate that an expectant approach is a reasonable option in the management of non-previa adherent placenta in carefully selected and highly motivated cases. Without PPH, there are no guidelines for the management of non-previa adherent RP, and available information is acquired from case reports and small case series. With conservative management of an adherent placenta, there is a substantial opportunity for spontaneous resorption and success in minimizing surgery-related complications while retaining fertility potential.

## Data Availability

The original contributions presented in the study are included in the article/supplementary material, further inquiries can be directed to the corresponding author.
